# Attenuated crosstalk between urothelium and fibroblasts promotes ureteral stricture development

**DOI:** 10.3389/fimmu.2026.1786116

**Published:** 2026-03-17

**Authors:** Rongchang Guo, Xuhong Zhang, Chengbang Wang, Junqi Cui, Kai Wang, Bao Hua, Shangqing Song, Yun Zou, Lin Zhou, Haisong Tan, Siyuan Liang, Le Tao, Jiangyi Wang, Wenfeng Li, Long Li, Guopeng Yu, Qing Yang, Yushan Liu, Bin Xu, Yiwei Wang

**Affiliations:** 1 Department of Urology, Shanghai Ninth People’s Hospital, Shanghai Jiao Tong University School of Medicine, Shanghai, China; 2 Department of Pathology, Shanghai Ninth People’s Hospital, Shanghai Jiao Tong University School of Medicine, Shanghai, China; 3 Department of Urology, The First People’s Hospital of Lancang Lahu Autonomous County, Yunnan, China

**Keywords:** fibroblast, single-cell RNA sequencing, ureter, ureteral stricture, urothelium

## Abstract

**Background:**

Ureteral stricture (US), characterized by fibrotic remodeling of the ureteral wall, represents an obstructive urological disorder with incompletely characterized pathophysiological mechanisms. This study integrates single-cell RNA sequencing (scRNA-seq) with immunohistochemical validation in human tissues to investigate the molecular and cellular mechanisms underlying US pathogenesis.

**Methods:**

Specimens of US (n = 7) and normal ureters (n = 8) were collected from patients prospectively. Single-cell RNA sequencing was performed to dissect the transcriptomic landscape of US, with subsequent immunohistochemical and immunofluorescence staining employed to validate key molecular and cellular findings at the protein level.

**Results:**

In US tissues, we identified significant downregulation of urothelial cell-specific gene signatures, accompanied by attenuated intercellular crosstalk between urothelial cells and fibroblasts. The urothelial cells exhibited reduced expression of reactive oxygen species (ROS)-associated functional clusters, with ANXA1 gene demonstrating particularly pronounced downregulation compared to control samples. Additionally, fibroblasts in US tissues displayed decreased expression of the THBS1 subtype and significant reduction in fibroblast-specific FPR2 receptor.

**Conclusions:**

Our findings establish that impaired urothelial cell function and disrupted urothelial-fibroblast communication are critically associated with or contributing to fibrotic remodeling in US. Specifically, control urothelial cells secrete ANXA1 as a ligand to interact with the fibroblast-expressed FPR2 receptor, maintaining fibroblast homeostasis. Clinically, these insights provide novel theoretical foundations for US prevention and highlight potential therapeutic targets for antifibrotic intervention.

## Introduction

Ureteral stricture (US) represents a prevalent and complex clinical entity in urology, characterized by abnormal narrowing of the ureteral lumen, which obstructs urinary drainage from the kidney to the bladder, leading to obstructive upper urinary tract pathology. When left undiagnosed or untreated, US may progress to chronic renal dysfunction, significantly compromising patient health and quality of life. Recent evidence has highlighted the crucial role of oxidative stress and inflammatory cascades in the progression of urinary tract injuries and subsequent renal impairment (1–3). The etiology of US is multifactorial, broadly categorized into congenital and acquired forms. Although their precise pathogenic mechanisms remain obscure (4, 5), congenital strictures (i.e., stenoses) primarily derive from ureteral structural anomalies during embryonic development, while acquired strictures encompass diverse causes, including impacted ureteral calculi, iatrogenic injury, trauma, inflammation or infection, radiation-induced damage (6), and immune-related disorders. Notably, with the widespread adoption of endoscopic urological techniques, the incidence of iatrogenic US has demonstrated an upward trend in recent years, given that the current management of US primarily involves two major approaches: endoscopic therapies and surgical interventions. Though endoscopic approaches (e.g., ureteral stenting and balloon dilation) effectively alleviate obstructive symptoms, they are often associated with complications such as hematuria, urinary tract infection, pain, and stricture recurrence (7, 8). Surgical intervention, while typically providing rapid obstruction relief, may entail substantial operative trauma and postoperative morbidities (9, 10).

Recently, advances in tissue engineering have offered new perspectives for US treatment, yet limited understanding of its molecular mechanisms has constrained clinical translation of tissue-engineered strategies (11). Although single-cell RNA sequencing (scRNA-seq) has been applied by Fink et al. to characterize cell types and signaling networks in healthy ureteral tissues (12), cellular alterations and pathological mechanisms underlying US remain uninvestigated.

scRNA-seq facilitates comprehensive cellular-resolution profiling of tissues, offering a versatile and rigorous methodological paradigm for dissecting the spatiotemporal molecular and cellular cascades underpinning disease pathogenesis and progression. This technology has been successfully applied in studying fibrotic diseases, encompassing renal (13), pulmonary (14), and dermal fibrosis (15). Leveraging scRNA-seq’s advantages in fibrosis research, this study systematically analyzed cellular heterogeneity and molecular mechanisms in US, with a focus on fibroblast-urothelial cell crosstalk. Thereby, these findings establish a theoretical framework for understanding US pathogenesis and identifying therapeutic targets.

## Results

### Histological characteristics of human ureteral stricture tissue and identification of major cell types by scRNA-seq

**Figure 1:**
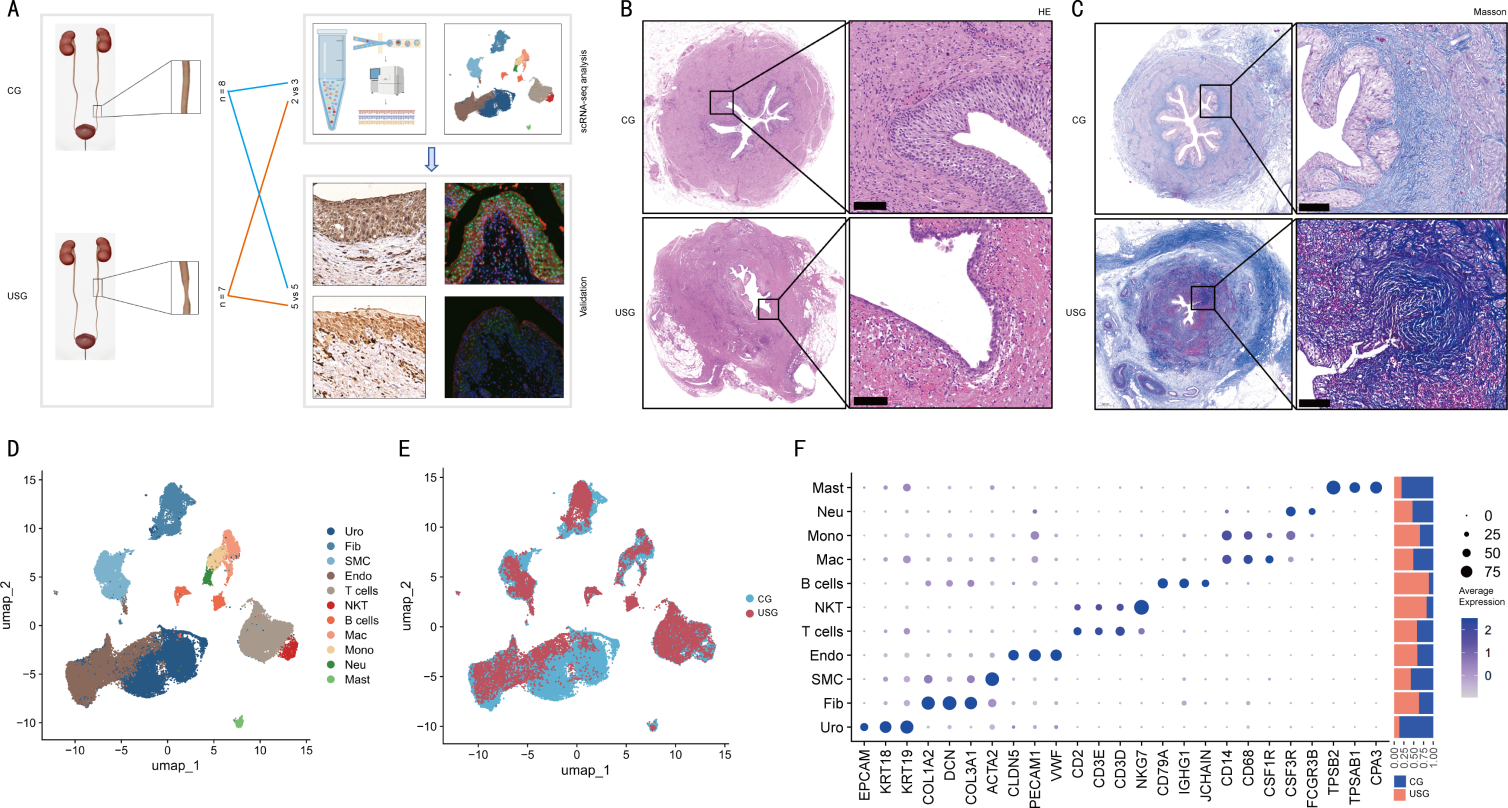
**(A)** Schematic diagram of sample collection, data analysis, and validation. Ureteral stricture group (USG) and control group (CG); **(B)** Representative HE staining images of control and stricture ureter tissues. Scale bar, 100 μm; **(C)** Representative Masson’s trichrome staining images of control and stricture ureter tissues. Scale bar, 100 μm; **(D)** UMAP visualization showing the 11 major cell clusters identified in the scRNA-seq dataset; **(E)** UMAP visualization illustrating the distribution differences between control and stricture ureteral cells in the scRNA-seq dataset; **(F)** Dot plot displaying the expression levels of representative marker genes in various cell types. Color intensity from light to dark indicates increasing gene expression levels, while dot size reflects the percentage of cells with non-zero expression of the respective gene. The right panel indicates the relative proportion of sample origins for each cell type.

To delineate the differences between US tissue and normal ureter tissue, we collected five surgical samples: two from the ureteral stricture group (USG) and three from the control group (CG). The tissues were processed into single-cell suspensions for scRNA-seq (16). Following data acquisition, comprehensive bioinformatic analyses were performed, with findings subsequently validated by histological staining of tissue sections (**Figure 1A**). HE and Masson’s trichrome staining revealed a significant reduction in urothelial cell density within the USG urothelium (**Figure 1B**), accompanied by marked increases in collagen fiber deposition in stricture regions compared with CG tissues (**Figure 1C**). To comprehensively profile the cellular composition in US, a total of 66,031 cells from both control and stricture ureter samples were isolated for scRNA-seq. After quality control, principal component analysis (PCA) was performed for dimensionality reduction, followed by graph-based clustering to group the 66,031 cells into 11 clusters, visualized via Uniform Manifold Approximation and Projection (UMAP). We identified 11 major cell types, including mast cells, neutrophils, monocytes, macrophages, B cells, natural killer T (NKT) cells, T cells, endothelial cells, smooth muscle cells, fibroblasts, and urothelial cells (**Figure 1D**). UMAP dimensionality reduction revealed distinct cellular distribution patterns between the two groups (**Figure 1E**). Cell types were annotated based on established biological markers, with their relative abundance shown in **Figure 1F**. Notably, urothelial cells and mast cells constituted a high proportion in control group tissues, while fibroblasts, natural killer T cells, and B cells were markedly increased in the US group tissues.

### Significantly reduced crosstalk between fibroblasts and injured urothelial cells

**Figure 2:**
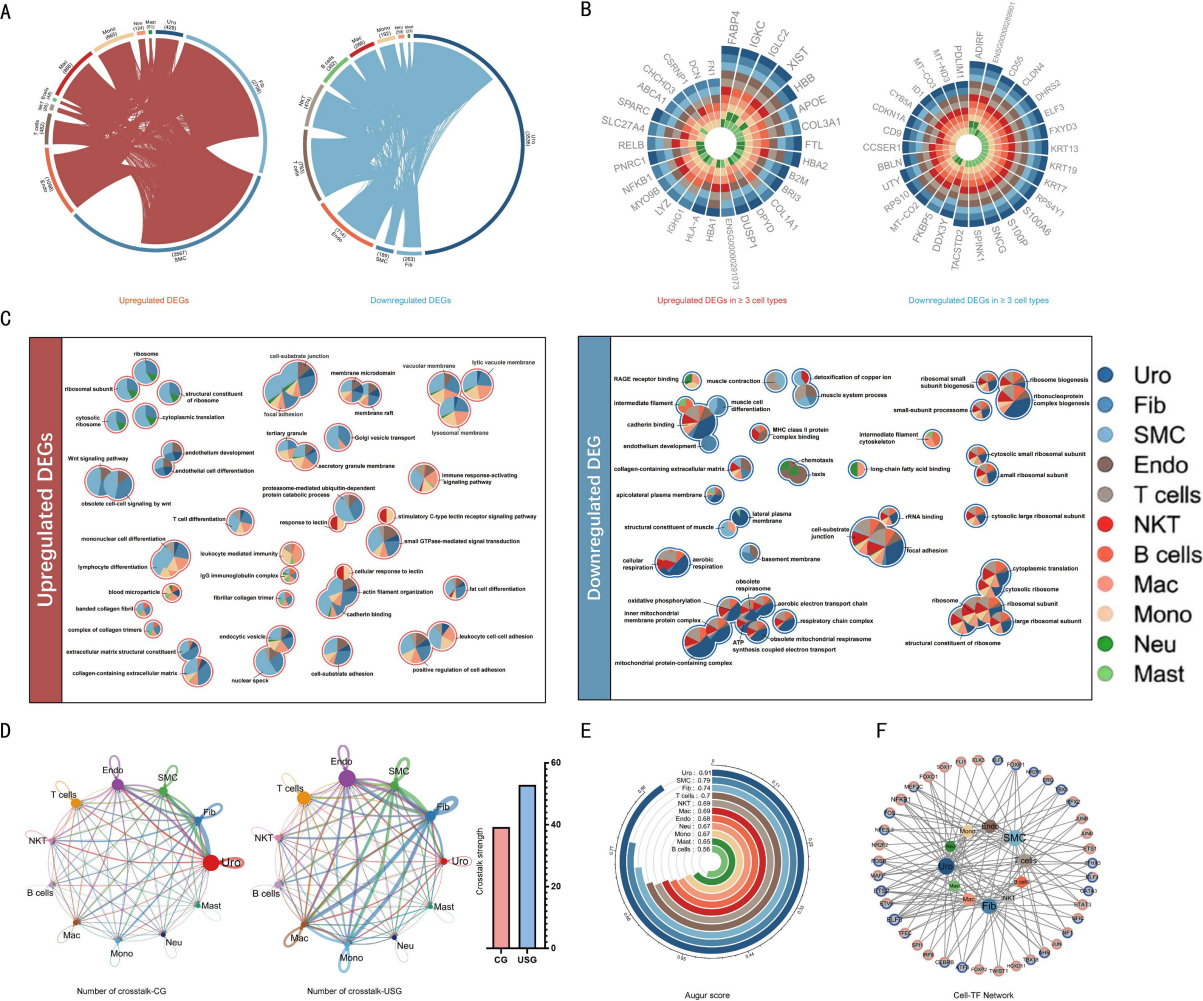
Single-cell analysis of ureteral stricture tissues. **(A)** Circos plot depicting upregulated and downregulated DEGs associated with ureteral stricture across cell types. Connecting arcs represent shared up/downregulated DEGs between cell types. **(B)** Radial plot illustrating DEGs (upregulated on left, downregulated on right) shared by ≥3 cell types. **(C)** Visualization network of representative GO terms and pathways for upregulated (left) and downregulated (right) DEGs in stricture tissues. Nodes represent GO terms/pathways, with internal pie charts showing gene proportion across cell types. **(D)** Circular plot displaying cell-cell crosstalk in ureteral tissues. Connecting lines indicate intercellular communication (thickness proportional to crosstalk frequency), with bar chart comparing total interaction strength between groups. **(E)** Arc plot of cell-type sensitivity scores to stricture microenvironments (higher scores represent greater sensitivity), with distribution shown across cell populations. **(F)** Cell type–transcription factor (TF) interaction network. Inner circular nodes represent cell types (size proportional to TF crosstalk frequency); outer ring nodes represent responsive TFs (orange/blue proportions indicate up/downregulated TF ratios).

A Circos plot was generated to visualize the overlap of differentially expressed genes (DEGs) across cell types based on single-cell analysis. The results revealed that smooth muscle cells (SMCs) and fibroblasts (Fibs) harbored the highest number of upregulated DEGs, with 3,567 and 2,708 genes respectively, while urothelial cells (Uro) accounted for nearly half of all downregulated DEGs (3,536 genes). These findings highlight SMCs, Fibs, and Uro cells as key contributors to US pathogenesis (**Figure 2A**). Fatty acid binding protein 4 (FABP4) was upregulated in 10 of 11 cell types, whereas COL3A1 (encoding type III collagen) was upregulated in 8 cell types. Conversely, CLDN4 (encoding claudin-4 tight junction protein) was downregulated across most cell types (**Figure 2B**).

Gene Ontology (GO) enrichment analysis of differentially expressed genes (DEGs) in ureteral stricture (US) tissues demonstrated significant enrichment of upregulated DEGs in four major functional categories: (1) protein biosynthesis(e.g., ribosomal structure and cytoplasmic translation), (2) cytoskeletal reorganization (e.g., actin filament polymerization and cell-matrix adhesion), (3) cell-matrix and cell-cell adhesion (e.g., focal adhesion complexes, extracellular matrix structural constituents, and positive regulation of cell adhesion), and (4) immune activation (e.g., leukocyte-mediated cytotoxicity, T cell differentiation, and immune response activation pathways). Pie chart quantification revealed cell-type-specific pathway distributions: immune-related pathways (particularly T cell differentiation and leukocyte-mediated immunity) were predominantly associated with immune cell populations (e.g., T lymphocytes, macrophages, and monocytes), while structural remodeling pathways (extracellular matrix organization and cytoskeletal rearrangement) were primarily enriched in smooth muscle cells, fibroblasts, and vascular endothelial cells. Notably, protein synthesis pathways showed dual enrichment patterns, with ribosomal biogenesis being most prominent in urothelial cells and translational machinery components also present in certain immune subsets (**Figure 2C**).

Comparatively, downregulated differentially expressed genes (DEGs) were primarily enriched in four principal categories (1) energy metabolism (e.g., cellular respiration, oxidative phosphorylation, mitochondrial electron transport chain), (2) protein biosynthesis with ribosome biogenesis, (3) muscle architecture and function (including muscle contraction and myocyte differentiation), and (4) cell adhesion. A pie chart analysis demonstrated that the repression of energy metabolic and protein synthetic pathways primarily occured in cell types with high structural and metabolic activity—smooth muscle cells, urothelial cells, and endothelial cells—suggesting functional suppression of these lineages in ureteral stricture (US) tissues.

Notably, downregulation of muscle system and cytoskeletal functions was most pronounced in smooth muscle cell populations. Concurrently, select immune and signaling pathways (e.g., MHC class II protein complex binding, RAGE receptor signaling) exhibited significant attenuation in macrophages and monocytes, implying remodeling of the local inflammatory regulatory network (**Figure 2C**).

From the cell-cell communication network diagram, urothelial cells and fibroblasts demonstrated crosstalk in control group tissues, whereas this interaction was diminished in US group tissues (**Figure 2D**). Augur analysis evidenced varying sensitivities among different cell types to the stricture environment, with urothelial cells (sensitivity score 0.91) showing the most pronounced response and representing the most sensitive cell population to the stricture condition. These findings exposed that urothelial cells might play a pivotal role and undergo significant alterations in stricture-associated pathological environments (**Figure 2E**).

Urothelial cells and fibroblasts performed extensive connections to transcription factors, with particularly pronounced TF regulatory activity (**Figure 2F**). These cell types served as major regulatory hubs in the network, as evidenced by their significant linkage to the highest number of TFs. Network analysis further revealed abundant representation of both upregulated (orange) and downregulated (blue) TF nodes. This presented that dynamic regulation by multiple TFs exerted a synergistic effect on pathological processes, including fibrosis and urothelial-mesenchymal crosstalk and remodeling, thereby collectively driving microenvironmental reconstruction.

### Atlas and differentiation trajectories of fibroblast subpopulations

**Figure 3:**
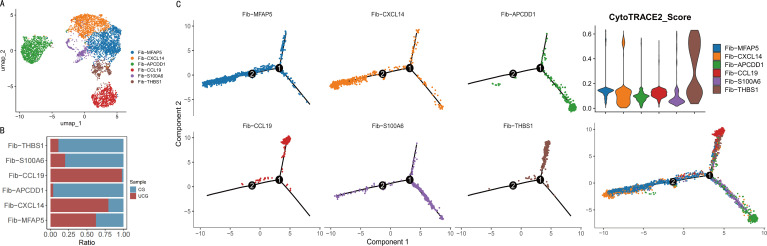
**(A)** UMAP plot showing the sample origins and distribution of fibroblast subpopulations; **(B)** Relative proportions of each fibroblast subpopulation; **(C)** Pseudotime analysis and differentiation trajectories of the fibroblast subpopulations, along with their differentiation potentials as indicated by CytoTRACE2 scores.

Via single-cell transcriptomics, we first characterized fibroblast composition and alterations, analyzing 6,803 fibroblasts (3,757 from the control group and 3,046 from the US group). Through UMAP dimensionality reduction and clustering, six major fibroblast subpopulations were clearly identified: Fib-MFAP5, Fib-CXCL14, Fib-APCDD1, Fib-S100A6, Fib-CCL19, and Fib-THBS1 (17–22). These subpopulations behaved clear separation in UMAP space, supported by gene expression heatmaps, demonstrating pronounced fibroblast heterogeneity (**Figure 3A**, **Supplementary Figure 1A**). Further analysis, i.e., subcluster distribution analysis, revealed marked differences between CG and USG. Specifically, Fib-CCL19, Fib-CXCL14, and Fib-MFAP5 were significantly enriched in the US group, while Fib-APCDD1, Fib-S100A6, Fib-THBS1 were more abundant in the control group (**Figure 3B**). Cell–cell interaction heatmaps depicted vigorous crosstalk between urothelial cells (Uro) and fibroblast subpopulations (Fib-MFAP5, Fib-CXCL14, Fib-APCDD1) in controls (**Supplementary Figure 1B**). In contrast, despite the general enhancement of intercellular crosstalk—especially within fibroblast subpopulations—the crosstalk between urothelial cells and multiple fibroblast subpopulations declined precipitously in the control group(**Supplementary Figure 1C**).

Next, a pseudotime analysis was performed to infer the differentiation trajectories of fibroblast subpopulations, yielding a branched trajectory structure where distinct subpopulations occupied specific positions along the pseudotime axis (**Figure 3C**). This revealed a dynamic transition process from early progenitor states to multiple differentiated endpoints. Fib-THBS1, Fib-CCL19, and Fib-APCDD1 were localized at the trajectory origin, indicative of high differentiation potential, whereas Fib-MFAP5 and Fib-CXCL14 resided at terminal branches, reflecting more mature or specialized functional phenotypes. CytoTRACE2 scoring further validated that Fib-THBS1 subpopulation harbored the highest differentiation potential among all clusters. Integrated analyses combining scRNA-seq and functional assays illustrated that Fib-THBS1 may employ unique biological functions and regulatory programs during fibroblast differentiation.

### Analysis of differential gene expression in urothelial cells

**Figure 4:**
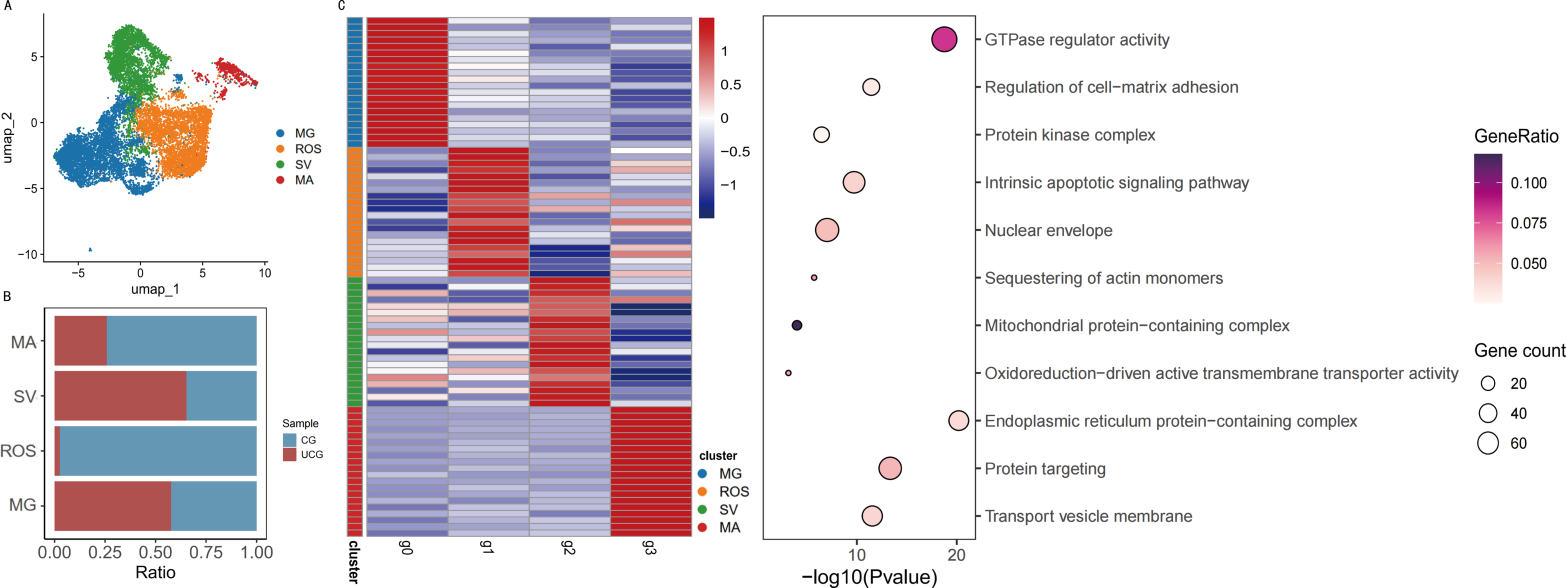
**(A)** UMAP visualization of the sample origins for each urothelial cell subpopulation; **(B)** the relative proportions of each urothelial cell subpopulation; and **(C)** the gene expression heatmap of the urothelial cell subpopulations (left), alongside the functional enrichment bubble plot of their differentially expressed genes (right).

The marked decrease observed in urothelial-fibroblasts crosstalk (**Figure 2D**) prompted a detailed investigation into urothelial cell composition and transcriptional alterations. A comprehensive analysis of 16,115 urothelial cells was performed, comprising 15,022 cells from control specimens and 1,093 cells from ureteral stricture (US) tissues (**Figure 4A**). Four distinct urothelial cell subtypes were identified based on their gene expression programs: migration-associated urothelial cells (MG), reactive oxygen species-responsive urothelial cells (ROS), secretory and vesicle-active urothelial cells (SV), and metabolically active urothelial cells (MA). Notably, the proportion of ROS-responsive urothelial cells was significantly higher in the control group yet scarcely detectable in the US group, manifesting that the ROS program underpinned the conferral of antioxidant defense capabilities to the urothelium (**Figure 4B**).

In the differential gene expression heatmap (**Figure 4C**), the vertical axis depicted significantly differentially expressed genes (DEGs), and the horizontal axis denoted distinct cell populations. The color gradient reflected gene expression levels, with red indicating high expression and blue indicating low expression. Each of the four UC subpopulations (MG, ROS, SV, and MA) exhibited discrete clusters of highly expressed genes, highlighting marked transcriptional heterogeneity among the subtypes. GO enrichment analysis of the DEGs, visualized via a bubble plot, revealed significant enrichment in pathways related to GTPase regulatory activity, cell-matrix adhesion, protein kinase complexes, mitochondrial functions, apoptosis, and transmembrane transport (**Figure 4C**). The DEGs among the UC subpopulations were primarily involved in essential biological processes, including cytoskeletal regulation, signal transduction, energy metabolism, and apoptosis.

Given the marked reduction in urothelial cell numbers, we hypothesized that upstream transcription factors might mediate this decline. Heatmap analysis of TF activity in ureteral urothelial cells uncovered that GATA3-uro development and KLF5-uro development were substantially enriched in control ureteral urothelial cells (**Supplementary Figure 1D**). To address this, we visualized the expression patterns of genes potentially associated with the transcription factors KLF5 and GATA3, as well as fibrosis-related genes, across different cell types (**Supplementary Figure 1E**). Through systematic gene mining and literature curation, we identified ANXA1 gene as exhibiting particularly strong fibroblast-associated expression patterns, consistent with prior reports demonstrating ANXA1’s role in fibroblast activation and tissue remodeling (22), which warranted further investigation.

### Downregulation of ANXA1 expression in urothelial cells and related immunological validation

**Figure 5:**
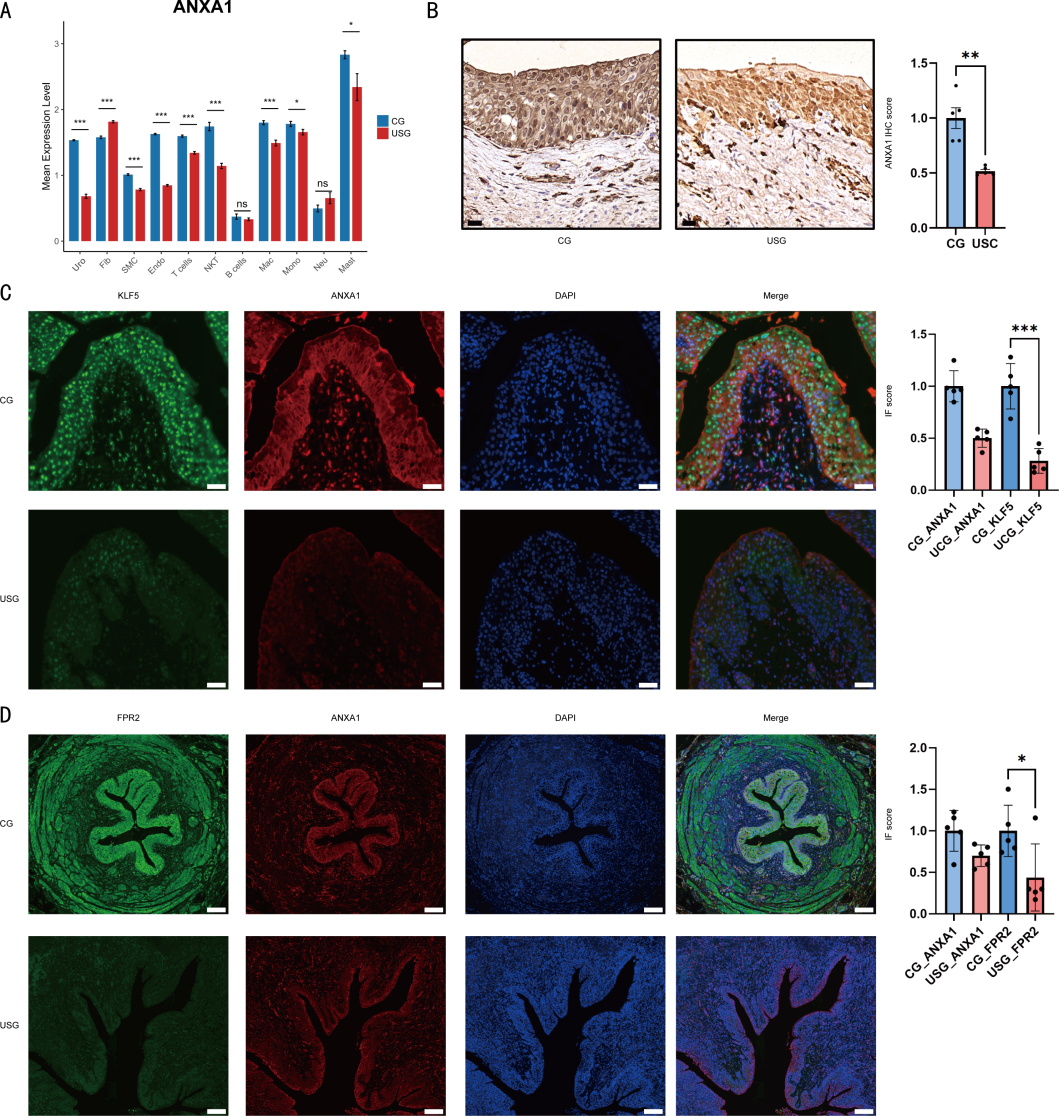
**(A)** Bar graphs illustrating the expression levels of the ANXA1 gene across eleven different cell types. Statistical significance is indicated as follows: ***p < 0.001 (highly significant), **p < 0.01 (very significant), *p < 0.05 (significant), and ns (not significant, p ≥ 0.05). **(B)** Immunohistochemical staining results of ANXA1 in tissue samples. Scale bar: 20 μm. **(C)** Co-expression immunofluorescence analysis of the transcription factor KLF5 and ANXA1 in CG and USG tissues, with quantitative analysis of fluorescent signals presented on the right. Scale bar: 50 μm. **(D)** Co-expression immunofluorescence results of ANXA1 and its receptor FPR2 in CG and USG tissues, with quantitative analysis of fluorescent signals shown on the right. Scale bar: 200 μm.

Through systematic evaluation of ANXA1 expression across eleven distinct cell populations, we identified significant downregulation in both urothelial and endothelial cell compartments (**Figure 5A**). To validate these transcriptomic findings at the protein level, we performed an immunohistochemical analysis, which evinced a pronounced ANXA1 depletion in US-derived urothelial cells relative to controls (**Figure 5B**). Subsequent dual immunofluorescence staining further supported these findings, demonstrating significant co-localization of the transcription factor KLF5 with ANXA1 (**Figure 5C**), as well as co-localization of ANXA1 with FPR2 (**Figure 5D**) within the tissues. Importantly, in the US group, the overall expression levels of KLF5, ANXA1, and FPR2 were all downregulated, suggesting that this molecular axis may be dysregulated during the development of US. Downregulation of KLF5, an upstream regulatory factor, may lead to decreased transcription of ANXA1, which in turn affects cellular function through the ANXA1-FPR2 pathway and contributes to the pathogenesis of US. Furthermore, immunofluorescence analysis confirmed the co-localization of the fibroblast marker DCN (12) and the FPR2 receptor within the same tissue regions (**Supplementary Figure 1F**).

## Discussion

Ureteral stricture (US), particularly when associated with impacted calculi and post-treatment restenosis, remains a significant clinical challenge in urology. Current management strategies predominantly involve surgical resection or endoluminal interventions. Notwithstanding short- to mid-segment US typically responds well to conventional surgical approaches, long-segment US often warrants complex reconstructive procedures with autologous tissue grafts, which are associated with substantial morbidity and impaired quality of life. Endoluminal therapies, primarily ureteral stenting, aim to maintain urinary tract patency. Recent investigations have focused on stent coating with antifibrotic agents (e.g., pirfenidone, rapamycin), aiming to locally inhibit fibroblast activation and extracellular matrix deposition at stricture sites. Preclinical studies in animal models have shown promising antifibrotic efficacy, with reduced fibrotic tissue formation (23–25). These advancements underscore the critical need to elucidate the molecular pathogenesis of US to identify novel therapeutic targets and inform the development of precision interventions. Systematic characterization of the cellular and molecular landscape of stenotic ureters will be essential for deciphering the mechanisms driving US progression and facilitating targeted therapeutic discovery.

Utilizing scRNA-seq technology, we comprehensively profiled 66,031 ureteral cells, providing an unbiased, single-cell resolution atlas of cellular dynamics during ureteral fibrosis. Our analysis revealed substantial remodeling of both cellular composition and functional states in US tissues. Histopathological evaluation via H&E and Masson’s trichrome staining demonstrated marked architectural disruption of the urothelial layer and excessive collagen deposition within stenotic regions in US specimens from patients who underwent repeated endourologic stone surgeries, establishing ureteral fibrosis—characterized by aberrant extracellular matrix (ECM) accumulation—as the core pathophysiological process driving US (26).

Building on these observations, we delved into the mechanisms underlying urothelial cell depletion and fibroblast-mediated extracellular matrix (ECM) overaccumulation. Single-cell sequencing data showed a significant reduction in urothelial cell proportion (**Figure 1F**) and functional impairment (**Figure 2A**) in stenotic tissues compared with controls, accompanied by markedly elevated fibroblast and smooth muscle cell activity (**Figure 2A**)—findings corroborated by histopathological staining. Our single-cell analysis revealed that smooth muscle cells (SMCs) harbored the highest number of upregulated DEGs (n=3,567), underscoring their central role in the pathogenesis of urethral stricture. This profound transcriptional hyperactivity likely reflects a pathological phenotypic switch from a contractile to a synthetic phenotype. Unlike healthy contractile SMCs, synthetic SMCs are characterized by high metabolic activity and the excessive secretion of extracellular matrix components, as evidenced by the widespread upregulation of collagen genes (e.g., COL3A1) observed in our dataset. Furthermore, the upregulation of FABP4 in SMCs suggests a metabolic reprogramming essential for sustaining the high energy demands of this proliferative state. This transition not only compromises the contractile function of the urethra but also actively contributes to the stiffening and narrowing of the urethral lumen.

Besides, intercellular communication analysis revealed robust urothelial-fibroblast crosstalk in normal ureters, which was profoundly attenuated in stenotic tissues (**Figure 2D**). Transcription factor interaction network analysis (**Figure 2F**) further indicated that both cell types were closely associated with several key transcription factors, manifesting a potent transcriptional activity. This comparative analysis raises the question of whether the weakened function of urothelial cells is closely tied to fibroblast activation. Drawing from findings in other organ fibrosis studies, the interaction between urothelial and stromal cells has been recognized as being associated with or contributing to the initiation and progression of fibrosis (13, 14). Taken together, these results suggest that disruptions in urothelial-stromal crosstalk may serve a critical function in ureteral fibrosis.

To gain deeper insights into the roles of urothelial and fibroblast cells in ureteral fibrosis, we performed subpopulation analyses. Among fibroblast subpopulations, the THBS1+ subtype not only accounted for a higher proportion in control ureteral tissues but also exhibited greater differentiation potential.

The specific depletion of this subpopulation in US tissues offers critical insight into the dynamics of fibrotic progression. Although THBS1 is classically recognized for its anti-angiogenic properties (27), our trajectory and CytoTRACE2 analyses identify Fib−THBS1 as a high-potential progenitor subset residing at the origin of the fibroblast lineage. The paradoxical decrease of this population in the US group is therefore best understood not as a simple loss of anti-angiogenic function, but as the consumptive differentiation of these progenitors into downstream effector phenotypes. Specifically, the reduction in Fib−THBS1 cells correlates inversely with the expansion of terminally differentiated, pro-fibrotic clusters such as Fib−MFAP5 and Fib−CXCL14. This suggests a pathogenic mechanism where the homeostatic, quiescent progenitor pool is exhausted to sustain the accumulation of synthetic fibroblasts that drive ECM deposition and stricture formation. Furthermore, this depletion may exacerbate fibrotic progression by attenuating anti-angiogenic activity, which in turn provides a vascular niche for fibroblast expansion and accelerates extracellular matrix deposition.

Urothelial subpopulation analysis revealed that reactive oxygen species (ROS)-responsive urothelial cells were more abundant in the control group, whereas this subpopulation was strikingly depleted in stenotic tissues. This suggests that under physiological conditions, urothelial cells protect the ureter through antioxidant defense mechanisms. However, ureteral urothelial damage induced by urinary stone formation and related interventions significantly compromises this antioxidant capacity. The loss of this specialized antioxidant defense leaves the urothelium vulnerable to oxidative damage, a conclusion further supported by our GO enrichment analysis (**Figure 4C**). The significant enrichment of genes associated with ‘mitochondrial functions’ and ‘apoptosis’ in the remaining urothelial cells provides molecular evidence of ongoing oxidative stress injury, as mitochondrial dysfunction is a primary consequence of ROS accumulation and a well-known trigger for programmed cell death. As a result, this deficiency may render the ureter susceptible to intense physicochemical stimuli from urine, triggering inflammation and fibrosis.

Transcription factor profiling of urothelial cells identified KLF5 as a highly enriched regulator. As members of the Kruppel-like factor (KLF) family, KLF5 belongs to a phylogenetically conserved class of transcription factors that orchestrate the expression of genes governing cell proliferation, differentiation, migration, apoptosis, inflammatory responses, barrier function, and metabolic adaptation (28). This discovery prompted us to characterize KLF5 downstream targets. Through integrative literature mining and transcriptomic analysis, we identified ANXA1 as a key transcriptional target of the KLF family (22). Functionally, ANXA1 mediates anti-inflammatory and tissue repair processes via its N-terminal peptide, which undergoes proteolytic cleavage to activate downstream signaling (29).

Subsequent expression analysis revealed significant downregulation of ANXA1 in both urothelial and endothelial cells, consistent with the observed impairment of urothelial function. However, ANXA1 loss alone could not fully account for fibroblast hyperactivity. Literature review highlighted formyl peptide receptor (FPR), a validated inflammation regulator (30), as a potential mediator. Leoni et al. demonstrated that in intestinal tissues, ANXA1 acts as an endogenous FPR ligand, activating ROS-dependent signaling to promote urothelial wound healing (29)—a mechanism congruent with our findings. Chen et al. further showed that urothelial-fibroblast crosstalk via ANXA1-FPR drives esophageal cancer progression (22), while Rudman-Melnick et al. documented injury-induced enhancement of ligand-receptor signaling between urothelial and stromal cells in a unilateral ischemia-reperfusion murine model of AKI(acute kidney injury) (13). Collectively, these studies underscore the critical role of urothelial-stromal communication in tissue homeostasis (31, 32), which is biologically plausible given the shared mesodermal origin of urothelium and kidney—this evolutionary conservation may explain the molecular similarity in stromal cell regulation between ureter and renal tissues.

To this end, we propose that the KLF5-ANXA1-FPR2 axis maintains ureteral homeostasis. Immunofluorescence analysis revealed spatiotemporal co-expression of KLF5 and ANXA1 in epithelial cells, alongside ANXA1-FPR2 co-expression in fibroblasts. Notably, expression of the KLF5-ANXA1-FPR2 axis was significantly downregulated in ureteral stricture tissues compared with controls, suggesting dysregulation of this signaling module in stricture pathogenesis. Thus, we postulate that dysfunction of the ANXA1-FPR2 signaling axis compromises urothelial cell-mediated regulation of fibroblast homeostasis, leading to aberrant myofibroblast activation and providing a molecular mechanism for ureteral fibrosis progression. Therapeutically, pharmacological restoration of ANXA1-FPR2 signaling represents a promising strategy to suppress fibroblast hyperactivity and mitigate fibrosis-induced US. This hypothesis not only illuminates the molecular mechanisms underpinning ureteral fibrosis but also identifies translatable intervention targets with clinical relevance.

Despite these findings, several limitations of our study should be acknowledged. Firstly, the limited sample size (n=7 for the stricture group) prevented us from performing advanced statistical analyses to adjust for potential confounders. Secondly, while our study identifies the KLF5-ANXA1-FPR2 axis through scRNA-seq and histological analysis, we did not perform *in vitro* co-culture experiments to definitively establish the functional impact of urothelial-derived ANXA1 on fibroblast behavior. Although our findings are supported by existing literature in other tissues, further functional validation is required to confirm these mechanisms in the context of ureteral fibrosis. Larger cohort studies and integrated *in vitro*/*in vivo* assays will be the focus of our future research once the necessary resources are secured.

## Conclusions

Our findings demonstrate that the impairment of urothelial cell function and the diminished communication between urothelial cells and fibroblasts are closely associated with or contribute to the progression of ureteral fibrosis. Specifically, dysfunction of the ANXA1-FPR2 signaling axis disrupts fibroblast homeostasis, leading to aberrant activation and extracellular matrix deposition. These insights may inform novel therapeutic avenues for the pharmacological management of US (23, 24).

### Patients and methods

Ureteral stricture specimens were surgically harvested postoperatively from patients who underwent ureteroureterostomy (stricture group, n=7), whereas normal ureteral tissues were obtained from radical cystectomy procedures (control group, n=8) (**Figure 1A**). Detailed clinical characteristics of the stricture cohort, including age, sex, etiology, stricture location, and length, are provided in **Supplementary Table 1**. Specifically, this group included 5 females and 2 males, with stricture lengths ranging from 2 to 8 cm. The inclusion criteria for the stricture group comprised: (1) documented history of multiple sessions of ureteroscopic laser lithotripsy for urolithiasis; (2) absence of prior renal pelvic or urothelial carcinoma. Control tissues were derived from histopathologically normal ureteral segments excised during radical cystectomy, with only specimens exhibiting negative intraoperative frozen section margins included to ensure the exclusion of residual pathological changes and maintain the validity of the control cohort. Following meticulous dissection, ureteral samples were either suspended in formaldehyde for immunohistochemical (IHC) analysis (n=10) or preserved in fresh culture medium for single-cell RNA sequencing (scRNA-seq, n=5).

### Statistical analysis

Due to the small sample size (n=7), statistical analysis was primarily descriptive. Continuous variables (e.g., age, stricture length) are presented as individual values or medians with ranges. Categorical variables are presented as frequencies.

### Immunochemistry

For morphological evaluation of ureteral tissues, paraffin-embedded samples from patients with ureteral stricture group and control group were analyzed by way of hematoxylin and eosin (HE) staining, Masson’s trichrome staining, immunohistochemistry (IHC), and immunofluorescence staining. Imaging was performed with a Nikon DS-U3 microscope. For immunofluorescence procedures, deparaffinized slides were subjected to antigen retrieval by incubation in EDTA buffer (pH 8.0) for 15 minutes, followed by sequential PBS washing. Endogenous peroxidase activity was blocked by incubating slides in 3% hydrogen peroxide at room temperature in the dark for 25 minutes. Blocking was performed by covering the slides evenly with 3% BSA solution. After removing the blocking solution, slides were incubated with either anti-FPR2 antibody (Nanjing Biolead, BS78333, 1:1500) or anti-KLF5 antibody (Nanjing Biolead, AF7542, 1:1500) in a humid chamber at 4°C overnight. The next day, following PBS washing, an HRP-conjugated secondary antibody (Abcam, ab205718, 1:5000 dilution) was applied and incubated at room temperature for 50 minutes. After subsequent PBS rinses, 520-TSA (HISTOV, DFT52100, 1:1000 dilution) was added and incubated in the dark at room temperature for 10 minutes, followed by PBS washing. After removing residual reagents, anti-ANXA1 antibody (Nanjing Biolead, BS6070, 1:1200 dilution) was applied, and slides were incubated overnight at 4 °C in a humid chamber. The following day, after washing, an HRP-conjugated secondary antibody (Abcam, ab205718, 1:5000 dilution) was added and incubated at room temperature for 50 minutes. Following PBS washing, slides were incubated with 570-TSA (HISTOV, DFT57100, 1:1000 dilution) in the dark at room temperature for 10 minutes, followed by PBS rinses. For nuclear staining, slides were air-dried slightly before nuclear staining with DAPI (Wknow, WK00020) at room temperature in the dark for 5 minutes, followed by PBS washing. Finally, slides were mounted with anti-fade mounting medium (Wknow, WK11003). Upon fluorescence detection, DAPI-stained nuclei appeared blue under UV excitation, FPR2-positive signals displayed green fluorescence (520 nm), and ANXA1-positive signals exhibited red fluorescence (570 nm). Quantitative optical density analysis was performed with the aid of ImageJ v1.8, expressed as the ratio relative to DAPI staining. Statistical analysis was conducted via Student’s t-test in GraphPad Prism v10.4, with p < 0.05 considered statistically significant.

### Single-cell RNA sequencing

Upon arrival at the laboratory, specimens from the ureteral stricture cohort (n = 2) and control group (n = 3) were immediately rinsed and subjected to macroscopic evaluation to characterize fibrotic patterning in the pathological samples. The detailed protocol for single-cell suspension preparation is described in the Supplementary Materials.

### scRNA-seq data analysis

Data processing and analysis, including quality control, normalization, and clustering, were implemented via the Seurat package (version 5.2.0) (33). Low-quality cells were filtered based on the following criteria: cells with fewer than 200 or more than 8,000 detected genes, or those with mitochondrial gene content exceeding 20% (34). Data normalization and dimensionality reduction were achieved via “SCTransform”, “RunPCA”, and “RunUMAP” functions. Cell type annotation was performed by identifying cluster-specific markers via ‘FindAllMarkers’ (Wilcoxon rank-sum test, log_2_FC > 0.5, p < 0.01) and cross-referencing the established cell type markers. Differential expression analysis between PCa and BPH groups within each cell type was conducted with “FindMarkers” (min.pct = 0.1, P < 0.05, |log_2_FC| > 0.25).

### Functional analysis

Functional enrichment analysis of DEGs was performed by means of the clusterProfiler package (v4.12.0), interrogating both Gene Ontology (GO) terms and Kyoto Encyclopedia of Genes and Genomes (KEGG) pathways. Enriched terms were filtered by a significance threshold of P < 0.05, and the results were subsequently visualized using ggplot2 (v3.5.1).

### Transcriptional regulatory network analysis

Capitalizing on the Single-Cell Regulatory Network Inference and Clustering (SCENIC) pipeline (35), we inferred transcriptional regulatory networks through three sequential steps: (1) co-expression module inference, (2) transcription factor motif enrichment, and (3) regulon activity quantification.

First, gene regulatory modules were constructed from the raw gene expression matrix using “grn” function from the pySCENIC package (version 0.12.1) in Python (version 3.9), which infers co-expression relationships between transcription factors and candidate target genes. Next, the “ctx” function was applied to identify enriched cis-regulatory motifs within each module, confirming potential regulatory crosstalk. Finally, the “aucell” function was adopted to assess regulon activity in individual cells by computing the area under the recovery curve. The resulting transcriptional regulatory network was visualized using Cytoscape (version 3.10.3).

### Cell-cell communication analysis

Intercellular communication was investigated by employing CellChat (36), an R package specifically designed for inferring ligand-receptor crosstalk from scRNA-seq data. By analyzing ligand expression in one cell type and corresponding receptor expression in others, CellChat enabled reconstruction of signaling networks and identification of key pathways mediating intercellular crosstalk.

### Augur analysis in scRNA-seq data

To identify cell types most responsive to biological perturbations in US, we deployed the Augur R package (v1.0.3), a specialized tool for quantifying cellular sensitivity in scRNA-seq datasets. Augur employs a machine-learning framework to evaluate the separability of perturbed and unperturbed cells in high-dimensional expression space, enabling the identification of cell populations with augmented transcriptional responsiveness.We applied the “calculate_auc” function to a Seurat object annotated with cell type and condition labels (e.g., age or disease state), generating AUC-based priority scores for each cell type. Results were visualized using the circlize R package (v0.4.16), providing an intuitive depiction of cell type sensitivity across aging trajectories.

### Differentiation states prediction and pseudotime analysis

Differentiation states prediction and stemness status of scRNA-seq data were estimated through the R package CytoTRACE (version 0.3.3), a reliable computational framework that assigned scores to single cells based on gene expression counts. These scores indicated transcriptome diversity and thereby reflected relative developmental potential (37). CytoTRACE scores ranged from 0 to 1, where higher values denoted increased stemness or reduced differentiation, and vice versa. Additionally, pseudo-time trajectories of tumor cell subpopulations were reconstructed with the help of Monocle (version 2.32.0) (38).

## Data Availability

The data presented in the study are deposited in the Genome Sequence Archive (GSA-Human) in the National Genomics Data Center (NGDC), accession number HRA016234 (BioProject: PRJCA056153), publicly accessible at https://ngdc.cncb.ac.cn/gsa-human. (39, 40).
