# Supplementary material for: Attenuated crosstalk between urothelium and fibroblasts promotes ureteral stricture development
Source: Front Immunol. 2026 Mar 17;17:1786116. doi: 10.3389/fimmu.2026.1786116 (PMC13035763; doi:10.3389/fimmu.2026.1786116)
Supplement: Supplementary file 1 [file SupplementaryFile1.docx]

**SUPPLEMENTARY METHODS**

***Single-cell suspension***

The total time from severing the blood supply to harvesting the ureteral tissue specimen is strictly controlled to within 30 minutes. After sample collection, the specimen is washed twice under sterile conditions with pre-cooled RPMI 1640 medium containing 0.04% BSA. Fresh tissue is then quickly placed on ice for cooling and transferred from the operating room to the laboratory for further processing.In the laboratory, the specimen is washed with pre-cooled Dulbecco’s Phosphate-Buffered Saline (DPBS; 311–425-CL; WISENT) at 4°C and cut into small fragments of approximately 0.5 mm³. The minced tissue is transferred to centrifuge tubes and centrifuged at 300 g for 5 minutes at 4°C. The supernatant is discarded. A digestion solution containing 10 ml of type IV collagenase (1.0 mg/ml; 17104019; Gibco) and DNase I (0.2 mg/ml; 10104159001; Roche) is added to the tissue, followed by gentle manual mixing. Digestion is performed in a 37°C water bath for 30 minutes. The reaction is terminated by adding an equal volume of DPBS.The digested mixture is passed through a BD 40 μm cell strainer 1–2 times, followed by centrifugation at 300 g for 5 minutes at 4°C, and the supernatant is discarded. The cell pellet is resuspended in an appropriate medium, and an equal volume of erythrocyte lysis buffer (MACS, catalog no. 130-094-183) is added. After gentle mixing, the suspension is incubated at 4°C for 10 minutes to lyse red blood cells. After erythrocyte lysis, the mixture is centrifuged again at 300 g for 5 minutes, and the supernatant is discarded. The cell pellet is washed once with medium and centrifuged under the same conditions to ensure purity. Finally, the cell pellet is resuspended in 100 μl of medium.Cell concentration and viability are assessed using a Luna cell counter to ensure the quality of cells meets the requirements for subsequent experiments.

***Droplet-based scRNA-seq***

The single-cell RNA sequencing (scRNA-seq) library was constructed using the 10× Genomics Chromium Next GEM Single Cell 3ʹ Reagent Kits v3.1 (Catalog No. 1000268). Specifically, single-cell suspensions were mixed with an enzyme reaction mixture and Gel Beads, and loaded into the 10× Genomics Single Cell Chip A. Under the operation of the Chromium Controller, water-in-oil droplets (GEMs) were generated. Within the droplets, single-cell barcoding and reverse transcription reactions were completed. The droplets were then recovered, and cDNA was extracted and purified using Silane magnetic beads, followed by PCR amplification to obtain sufficient cDNA.The amplified cDNA was further purified with SPRIselect magnetic beads, and quality control was performed using the Agilent Bioanalyzer High Sensitivity Chip to ensure that the main fragment size distribution fell within the range of 400-600 bp. Subsequently, library construction was carried out following the standard workflow outlined in the 10× Genomics User Guide, including fragmentation, end repair, adapter ligation, and sample index PCR steps. This procedure ultimately resulted in the generation of a sequencing-ready library. The entire experimental process strictly adhered to the official 10× Genomics protocols.In this study, scRNA-seq data from ureteral samples underwent standardized analysis. First, raw Fastq files were processed using Cell Ranger software (version 5.0.0, 10× Genomics), and sequence data were aligned to the human reference genome GRCh38. Cell Ranger precisely quantified sequencing data by identifying cell barcodes and unique molecular identifiers (UMIs), and generated quality control metrics such as the number of high-quality cells, median gene expression, and sequencing saturation levels.
